# Supplementary material for: Chromatographic Scalable Method to Isolate Engineered Extracellular Vesicles Derived from Mesenchymal Stem Cells for the Treatment of Liver Fibrosis in Mice
Source: Int J Mol Sci. 2023 May 31;24(11):9586. doi: 10.3390/ijms24119586 (PMC10253331; doi:10.3390/ijms24119586)
Supplement: Supplementary file 1 [file ijms-24-09586-s001.zip › ijms-2410371-supplementary.pdf]

Chromatographic Scalable Method to Isolate Engineered Extracellular Vesicles Derived from Mesenchymal Stem Cells for the Treatment of Liver Fibrosis in Mice

Luciana M. Domínguez [1], Bárbara Bueloni [1], Ma. José Cantero [1], Milagros Albornoz [1], Natalia Pacienza [2], Celeste Biani [3], Carlos Luzzani [3], Santiago Miriuka [3], Mariana García [1], Catalina Atorrasagasti [1], Gustavo Yannarelli [2], Juan Bayo [1], Esteban Fiore [1,*,†] and Guillermo Mazzolini [1,4,*,†]

[1] Laboratorio de Terapia Génica, Instituto de Investigaciones en Medicina Traslacional (IIMT), Universidad Austral—CONICET, Pilar B1629, Buenos Aires, Argentina
[2] Instituto de Medicina Traslacional, Trasplante y Bioingeniería (IMeTTyB), Universidad Favaloro-CONICET, Ciudad Autónoma de Buenos Aires C1078, Argentina
[3] LIAN-CONICET, Fleni, Belén de Escobar B1625, Buenos Aires, Argentina
[4] Liver Unit, Hospital Universitario Austral, Universidad Austral—CONICET, Pilar B1629, Buenos Aires, Argentina
* Correspondence: efiore@austral.edu.ar (E.F.); gmazzoli@austral.edu.ar (G.M.)
† These authors contributed equally to this work.

Supplementary Figures

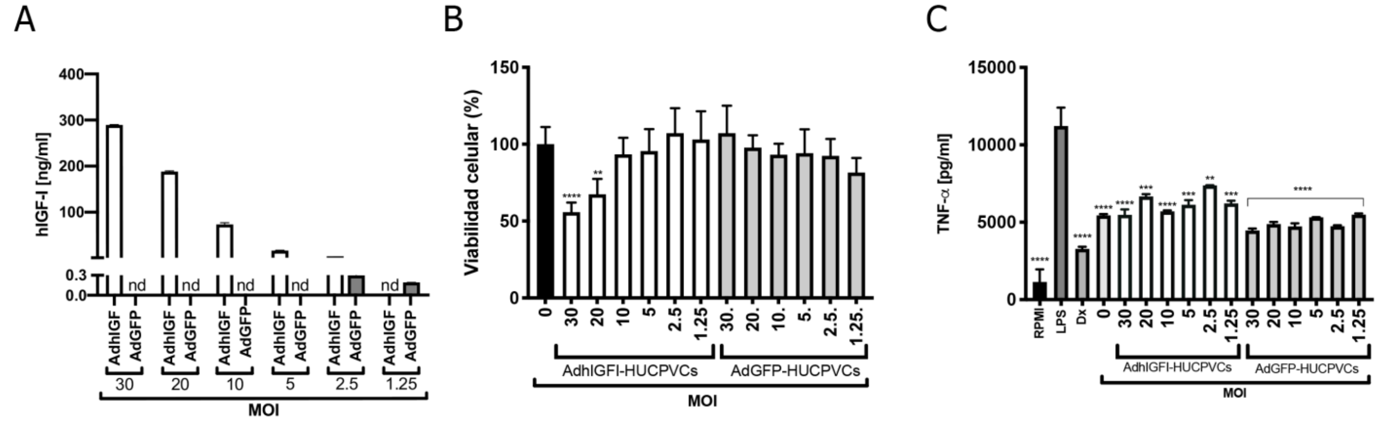

Supplementary Figure S1: Setting HUCPVCs infection with AdhIGF-I to load IGF-I on EVs. HUCPVCs were infected with increasing amounts of AdhIGF-I or AdGFP, expressed as multiplicity of infection (MOI). (**A**) IGF-I level on HUCPVCs supernatants (sn) 3 days after infection with AdhIGF-I (white bars) or AdGFP (gray bars) determined by ELISA. (**B**) Cell viability determined by MTT assay. Black bars represent without infection condition. **p<0.01; **** $p < 0.0001$ vs. without infection. (**C**) Anti-inflammatory capacity of AdhIGFI-HUCPVCs. J774 macrophages cell line (Mσ) were co-incubated 4 hours with LPS and supernatants of AdhIGF-I or AdGFP infected HUCPVCs at increasing MOI. Non-stimulated macrophages by LPS

(gray bar) and co-incubation with LPS and dexamethasone were used as assay control. TNF-α levels on J774 cells sn were determined by ELISA. ** $p < 0.01$; *** $p < 0.001$; **** $p < 0.0001$ vs. LPS.

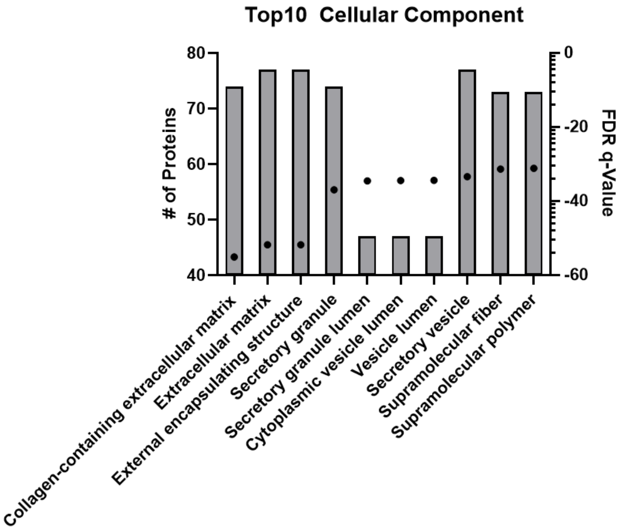

Supplementary Figure S2: Top 10 “Biological Component” of GO analysis of co-expressed proteins on AdhIGF-I-HUCPVC-EVs, AdGFP-HUCPVC-EVs and HUCPVC-EVs. Graph shows number of genes (right axis, column bars) and Q-value B&H (left axis, dots). FDR, false discovery rate.

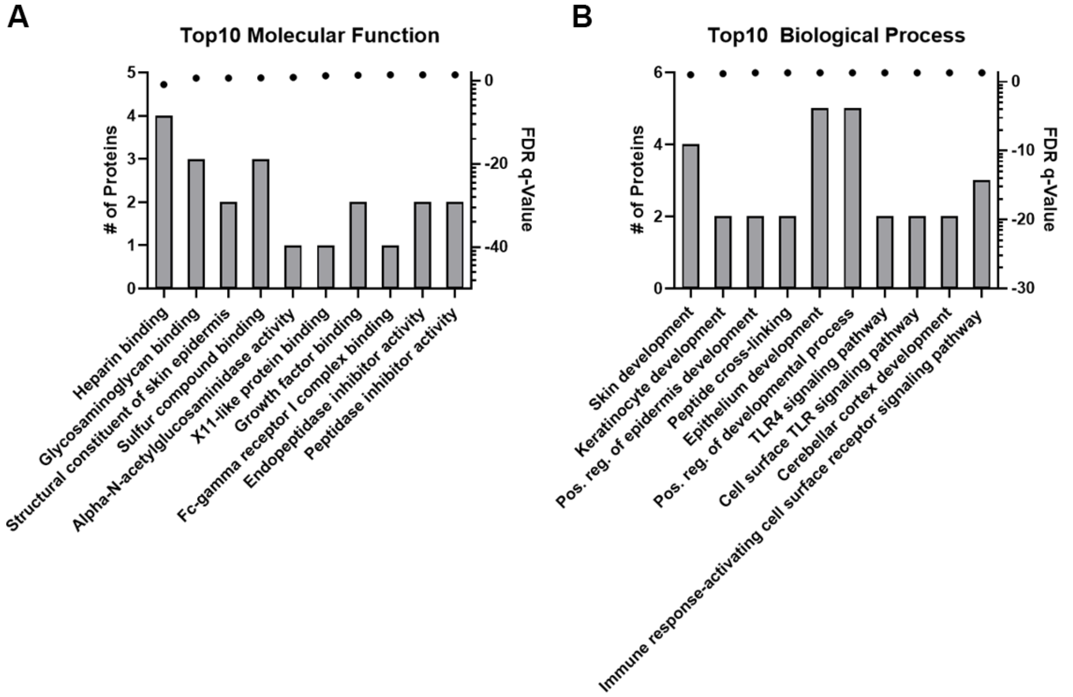

Supplementary Figure S3: Top 10 “Molecular Function” (**A**) and “Biological Process” (**B**) of GO analysis of over-represented proteins on AdhIGF-I-HUCPVC-EVs in comparison with HUCPVC-EVs. Graph shows number of genes (right axis, column bars) and Q-value B&H (left axis, dots). FDR, false discovery rate.

Supplementary Table S1: Proteins identified and intersection on Venn diagram of AdhIGF-I-HUCPVC-EVs, AdGFP-HUCPVC-EVs and HUCPVC-EVs.

**EVs condition**	**Proteins**
AdhIGF-I-HUCPVC-EVs & HUCPVC-EVs & AdGFP-HUCPVC-Evs Total=243	SEC23A
	FSTL1
	ACTN4
	ACTB
	MMP2
	PSMA2
	ANXA2
	PSMA4
	KRT19
	LTBP2
	ENO1
	3 SV
	HSPB1
	CCT4
	COL3A1
	SERPINE1
	LCN1
	LAMA2
	AP2M1
	KRT14
	GC
	LUM
	ANXA6
	C1S
	TUBA4A
	ANXA1
	C1R
	AP2A1
	TLN1
	CLSTN1
	EEF2
	CTSB
	NID1
	KRT9
	WARS1
	JUP
	COL6A2
	FBLN1
	FLT1
	EDIL3
	HSPA13
	GANAB
	LOX
	GSN
	TNC
	PDIA6
	YWHAG
	PTGFRN
	MYH14

**EVs condition**	**Proteins**
AdhIGF-I-HUCPVC-EVs & HUCPVC-EVs & AdGFP-HUCPVC-EVs	DSP
	DPYSL2
	KRT77
	SERPINH1
	ITGB1
	KRT76
	KRT80
	KRT27
	AMY1A
	ATP5F1B
	TUBA1A
	COL5A2
	PSMB2
	PKM
	PCOLCE
	VIM
	UBA1
	SERPINF1
	LGALS3BP
	LYZ
	PSAP
	FASN
	PIP
	OLFML3
	ITGA3
	INHBA
	LDHA
	PSMA3
	TGM2
	SERPINE2
	QSOX1
	PSMA1
	CAND1
	MFGE8
	A2M
	KRT1
	LAMB1
	COL6A3
	LOXL2
	MYH10
	CASP14
	COL1A1
	PDIA3
	LRP1
	POSTN
	KRT10
	CCT5
	TALDO1
	NAGLU

**EVs condition**	**Proteins**
AdhIGF-I-HUCPVC-EVs & HUCPVC-EVs & AdGFP-HUCPVC-EVs	ARG1
	KRT4
	GARS1
	FBN2
	TPP1
	P3H1
	KRT13
	MANBA
	KRT5
	TIMP2
	H3-3B
	HEXB
	EIF4A1
	PLTP
	FSCN1
	P4HB
	FLNB
	STC2
	PDCD6IP
	COL6A1
	AP2B1
	WDR1
	C3
	ACLY
	RAB10
	KRT6B
	FLG2
	PSMB1
	SPARC
	CLEC11A
	COL5A1
	PSMA6
	HSPA5
	ALB
	TUBB
	THBS3
	SERPINC1
	CCDC80
	DEFA1
	PSMB6
	LMNA
	ACTN1
	PTX3
	KPRP
	KPNB1
	CLU
	DSC1
	CCT2
	EMILIN1

EVs condition	Proteins
AdhIGF-I-HUCPVC-EVs & HUCPVC-EVs & AdGFP-HUCPVC-EVs	MYH9
	DSG1
	PXDN
	GAPDH
	KRT16
	CTSD
	KRT84
	CD63
	SERPINB3
	GLB1
	EFEMP2
	GAS6
	RPS27A
	RPS3
	THBS1
	NUCB1
	LDHB
	KRT31
	AEBP1
	IGHA1
	PSMA7
	PLS3
	FBN1
	BMP1
	TGFBI
	PLOD3
	ACTC1
	ITIH2
	S100A8
	PIGR
	PRDX1
	PLOD2
	HEXA
	THBS2
	LTF
	ZG16B
	SERPINB12
	HSP90AB1
	DYNC1H1
	H2BC15
	FLNC
	VCP
	CCT3
	KRT71
	RPS16
	ALDOA
	KRT78
	PZP
	HSP90B1

EVs condition	Proteins
AdhIGF-I-HUCPVC-EVs & HUCPVC-EVs & AdGFP-HUCPVC-EVs	FLNA
	PSMB5
	C4A
	IGHG1
	KRT6A
	COL1A2
	PAPPA
	CLIC1
	H4C1
	PSMA5
	KRT18
	APOM
	HSPA8
	LAMC1
	CLTC
	HTRA1
	ANXA5
	CRTAP
	SHMT2
	KRT86
	RAN
	COL12A1
	SLC2A1
	EEF1G
	EEF1A1
	MVP
	AFP
	CD109
	HBB
	SPON2
	VASN
	KRT75
	FN1
	KRT17
	PLSCR3
	HSP90AA1
	FBLN5
	EFEMP1
	RAB7A
	S100A9
	PGK1
	YWHAZ
	HBA1
	KRT2
	PLOD1
	DCD
	HRNR

EVs condition	Total	Proteins
AdhIGF-I-HUCPVC-EVs & HUCPVC-Evs	6	
		ILF2
		VAT1
		PDIA4
		POTEE
		CCT7
AdhIGF-I-HUCPVC-EVs & AdGFP-HUCPVC-EVs	8	KRT82
		PKP1
		MEGF8
		KRT33A
		KRT85
		QPCT
		KRT34
		ECM1
HUCPVC-EVs & AdGFP-HUCPVC-EVs	17	RPLP0
		NIBAN2
		IQGAP1
		HYOU1
		KRT38
		KRT12
		SEPTIN2
		FBLN2
		CFH
		PLEC
		NME1
		NID2
		HSPA4
		RPSA
		DPYSL3
		AHCY
		KRT32
AdhIGF-I-HUCPVC-Evs	4	TTYH3
		PSMD11
		KRT33B
		KRT3
HUCPVC-Evs	4	1 SV
		PYGB
		YWHAQ
		CCT8
AdGFP-HUCPVC-EVs	1	PLAT

Supplementary Table S2: List of proteins of Volcano Plot analysis.

Differential expression analysis AdhIGF-I-HUCPVC-EVs vs HUCPVC-EVs	
NAGLU	Non-significative regulated proteins
SERPINE2	
COL5A1	
IGHG1	
LTF	
CLSTN1	
KRT2	
KRT10	
VASN	
HYOU1	
KRT18	Upregulated proteins
PTX3	
S100A8	
CD109	
AMY1A	
KRT27	
PIGR	
IGHA1	
PSMA6	Downregulated proteins
PSMB2	
PSMA1	
PSMB1	
PSMA2	
RPS16	
PSMB6	
PSMB5	

Differential expression analysis AdhIGF-I-HUCPVC-EVs vs AdGFP-HUCPVC-EVs	
PSAP	Non-significative regulated proteins
CTSB	
PSMA6	
CLU	
PSMA4	
PSMB5	Downregulated proteins
KRT84	
INHBA	
KPNB1	
KRT19	
PSMB1	
VCP	
RPS16	
PSMA2	
PSMA1	
RAB7A	
YWHAZ	
PSMB2	
PSMB6	
PSMA5	
PSMA7	
VIM	

Supplementary Material And Methods

EVs characterization

Particle size and morphology of EVs were characterized using transmission electron microscopy (TEM) [1]. Briefly, an aliquot of EVs suspension was loaded into copper-coated grids and incubated at room temperature for 10 minutes. After 3 washes in ultrapure water, the sample was negatively stained with 2% sodium phosphotungstate for 30 seconds. Staining solution was removed using filter paper and the grid was stored at room temperature in the dark until imaging. All the samples were observed in a JEM 1200EXII transmission electron microscope (JEOL, Japan) at 80 kV.

Flow cytometry was used to detect CD9, CD63 and CD81 surface markers. In brief, 50 µl of individual o pooled fraction was bound with 5 µl of anti-CD63 antibody-coated magnetic beads (Life Technologies, Norway), as recommended by the manufacturer. Next, anti-CD63 bounded EVs were stained with anti-CD81-PE and anti-CD9-APC antibodies (Molecular Probes, Life Technologies, OR, USA) for 30 min at room temperature. Beads were then washed with PBS 0.1% BSA and were analyzed BD Accuri cytometer. At least 10000 events per treatment were counted.

Size distribution particles was performed by Microfluidics Resistive Pulse Sensing (MRPS) measurements using the nCS1 instrument (Spectradyne, Torrance, CA) in the Functional Genomics Unit, Institut Pasteur de Montevideo, Montevideo, Uruguay [2]

Cell viability assay for adenoviral infected HUCPVCs

For cell viability assays, HUCPVCs at 70% of confluence in 96 well plates and the next day infected with increasing MOI of AdhIGFI or AdGFP. After 3 days, cells viability was assessed by standard MTS assays using Promega’s Cell Titer reagents according to the manufacturer’s protocols. Absorbance at 490 nm and 650 nm (reference wavelength) measured by a FlouroStar Omega (BMG Biosciences) plate reader. Data were normalized to the uninfected HUCPVCs (100% viability). Three independent experiments were performed.

In Vitro macrophage assay

The analysis of HUCPVCs anti-inflammatory potential was performed using a protocol adapted from *Pacienzia et al.* [3] Briefly, the macrophage cell line J774.1 (kindly provided by Juan Gallo, UA, Argentina) was seeded (2x10E5 cells/well) on 12-well plate with culturing medium (RPMI, 10% FBS, 100 U/mL penicillin and 100 mg/mL streptomycin). After 20 h of incubation, the J774.1 cells were stimulated by replacing the culturing medium with a similar volume of medium alone (10% FBS and antibiotics) or containing LPS (10 ng/mL), LPS plus dexamethasone (1 mg/mL), or LPS in combination with CM of HUCPVCs, AdGFP-HUCPVCs or AdhIGFI-HUCPVCs (diluted at 50%). All the different conditions were prepared in a total volume of 1 mL and tested in triplicates. The conditioned medium was collected after 4 h of incubation at 37°C and kept frozen until assayed for TNF-α by ELISA (BD OptEIA™ Set Mouse TNF- α, BD Bioscience, CA, USA) following the manufacturer's recommendations. The protein levels determinations were performed in duplicates, in two or three independent experiments.

IGF-I quantification

Human IGF-I was measured by ELISA (R&D System, MN, USA) following manufacturers´ protocol. CM or chromatography fractions of HUCPVCs and AdhIGFI-HUCPVCs was applied directly to the wells.

To evaluate the presence of IGF-I within the EVs, protein levels in EVs derived from HUCPVCs and AdhIGFI-HUCPVCs, lysates or non-lysates, were compared by ELISA (R&D System, MN, USA). The lysis of the EVs was done with buffer 1% Triton-X100, 0,1% SDS in H_2O. Dialysis of EVs preparation was performed with a 300 kDa cut-off membrane (Spectrum™ Labs) against PBS. EVs preparations was diluted 1/10 to 500 μl of final volume and dialyzed against 1000 ml of PBS for 4 h in agitation at 4 °C. Then, buffer was replaced with 1000ml of fresh PBS and incubated overnight in agitation at 4 °C.

Proteomic analysis of EVs derived from HUCPVCs

Mass spectrometry analysis: Liquid chromatography tandem mass spectrometry (LC-MS/MS) was performed on three biological replicates of EVs derived from HUCPVCs, AdGFP-HUCPVCs or AdhIGF-I-HUCPVCs. EVs were concentrated by ultracentrifugation for 2 h at 100.000× *g* and then lysed in RIPA

buffer. Protein digestion and mass spectrometry analysis were performed at the Proteomics Core Facility CEQUIBIEM, at the University of Buenos Aires, Argentina as follows: the protein samples were reduced with dithiothreitol in 50mM of ammonium bicarbonate at a final concentration of 10mM (45 min, 56 °C) and were alkylated with iodoacetamide in the same solvent at a final concentration of 20mM (40 min, room temperature (RT), in darkness). This protein solution was precipitated with 1/5 volumes of trichloroacetic acid at 20°C for at least 2 h and was centrifuged at maximum speed for 10 min (4 °C). The pellet was washed twice with cool acetone and was dried at RT. The proteins were resuspended in ammonium bicarbonate 50 mM, pH = 8, and were digested using trypsin (V5111; Promega, Madison, WI, USA). Next, the peptides were purified and desalted via ZipTip C18 columns (Millipore, Burlington, MA, USA). The digests were analyzed via nanoLC-MS/MS in a Thermo Scientific Q-Exactive Mass Spectrometer coupled to a nanoHPLC EASY-nLC 1000 (Thermo Scientific). For the LC-MS/MS analysis, approximately 1 µg of peptides was loaded onto the column and was eluted for 120 min using a reversed-phase column (C18, 2 µm, 100 A, 50 µm x 150 mm) Easy-Spray Column PepMap RSLC (P/NES801) that was suitable for separating protein complexes with a high degree of resolution. The flow rate used for the nanocolumn was 300 nl min1, and the solvent range used was from 7% B (5 min) to 35% B (120 min). Solvent A was 0.1% formic acid in water, whereas B was 0.1% formic acid in acetonitrile. The injection volume was 2 µL. The MS equipment has a high collision dissociation cell (HCD) for fragmentation and an Orbitrap analyzer (Q-Exactive, Thermo Scientific). A voltage of 3.5 kV was used for electrospray ionization (Thermo Scientific, EASYSPRAY). XCalibur 3.0.63 software (Thermo Scientific) was used for data acquisition and equipment configuration that allows peptide identification simultaneously with their chromatographic separation. Full-scan mass spectra were acquired in the Orbitrap analyzer. The scanned mass range was 400–1800m/z, at a resolution of 70,000 at 400 m/z, and the 12 most intense ions in each cycle were sequentially isolated, fragmented by HCD, and measured in the Orbitrap analyzer. Peptides with a charge of +1 or with unassigned charge state were excluded from fragmentation for MS2.

Analysis of MS data: Q-Exactive raw data was processed using Proteome Discoverer software (version 2.1.1.21, Thermo Scientific) and was searched against *Homo sapiens* protein sequence database with trypsin specificity and a maximum of one missed cleavage per peptide. Carbamidomethylation of cysteine residues

was set as a fixed modification, and oxidation of methionine was set as variable modification. Proteome Discoverer searches were performed with a precursor mass tolerance of 10 ppm and product ion tolerance of 0.05 Da. Static modifications were set to carbamidomethylation of Cys, and dynamic modifications were set to oxidation of Met and N-terminal acetylation. Protein hits were filtered for high confidence peptide matches with a maximum protein and peptide false discovery rate of 1%, which was calculated by employing a reverse database strategy. Proteome Discoverer then calculates the Peptide Spectrum Matches (PSMs) for each protein in each condition. This parameter represents the total numbers of peptides identified for a certain protein.

Statics and Bioinformatic analysis: A label-free semi-quantitative analysis of protein abundance was performed using the PSMs calculated by Proteome Discoverer. Bioinformatic analysis was performed in Perseus v.1.6.2.3. Proteins with only one valid value in each condition, in only one condition, or with missing values in all three replicates in each of the three conditions were filtered out, selecting proteins with at least two valid values in at least one condition. Next, log2 transformation was performed and missing values (NaN, “Not a number”) were imputed by the minimum detected values of the normal distribution of the whole dataset (Width = 0.5, Down shift = 1.8). Next, the abundance averages for each protein were compared between treatments through the t-test. Proteins were considered significantly regulated when: (A) The t-test showed a value of significance $p < 0.05$, (B) the value of the difference between the log-scale averages of abundance (log2) was above or below the fold change (FC) 1 and −1 ($-1 < FC$ difference < 1), respectively. Gene ontology and pathway analysis were performed using the ToppGene suite [4].

Reverse transcription-polymerase chain reaction (RT-PCR) and qPCR

Total RNA was extracted by using Trizol Reagent (Sigma-Aldrich, MO, USA) from EVs, cells, or tissue. Total RNA was reverse transcribed with 200U of Super- Script II Reverse Transcriptase (Life Technologies, CA, USA) using 500 ng of Oligo (dT) primers. cDNAs were subjected to real-time polymerase chain reaction (qPCR) (Stratagene Mx3005p, Stratagene, CA, USA). The mRNA levels were quantified by SYBR® Green (Life Technologies, CA, USA): COL1A2 and α-SMA according to the experiment; using the following primers: COL1A2 forward 5’-CCTACATGGACCAGCAGACTG-3’ and reverse 5’-

GGAGGTCTTGGTGGTTTTGTA-3'; α-SMA forward 5'-ACTGGGACGACATGG AAAA-3' and reverse 5'-CCATCTCCAGAGTCCAGCAC-3'. All PCR amplifications were carried out using a cycle of 95 °C for 10 min and 40 cycles under the following parameters: 95 °C for 30 s, corresponding melting temperature for 1 min, 72 °C for 1 min. At the end of the PCR reaction, the temperature was increased from 60 °C to 95 °C at a rate of 2 °C/min, and the fluorescence was measured every 15 s to construct the melting curve. Values were normalized to levels of glyceraldehyde-3-phosphate dehydrogenase (GAPDH; used as housekeeping; forward 5'-CATCTCTGCCCCCTCTGCTG-3' and reverse 5'-GCCTGCTTCACCACCTTCTTG-3') transcript. Data were processed by the ΔΔCt method. The relative amount of the PCR product amplified from untreated animals (saline solution or DMEM condition). A non-template control was run in every assay, and all determinations were performed in triplicates, in two or three independent experiments.

Sirius red staining and immunohistochemistry

Formalin-fixed liver samples were paraffin-embedded and serially sectioned at 5 μm thickness. Paraffin was removed from slides with xylene followed by graded ethanol solutions and rehydration on PBS. For fibrillary collagen detection, sections were stained with Sirius Red for 1 hour. Quantitative analysis of stained area was performed by computerized morphometric analysis. About 50 light microscope images (×200) per specimen were captured and analyzed using the color threshold detection system developed in ImageJ software (NIH, USA). Results were expressed as percentage of positive area.

For α-SMA and PCNA immunostaining, endogenous peroxidase was blocked by incubating the preparations with 3% Hydrogen Peroxide in Methanol for 30 minutes. Antigenic retrieval was performed in pH 6.0 citrate buffer by heating the sample in microwaves. Avidin and endogenous biotin were blocked by incubating with the respective blocking solutions (Vector Laboratories, Inc. USA). Polyclonal anti-α-SMA (1/100; Abcam Inc., USA) or monoclonal anti-PCNA (1/100; Santa Cruz Biotechnology, USA) was applied overnight at 4°C. After washing, slides were incubated at room temperature for 2 hours with biotinylated goat anti-rabbit (1/100, Vector Laboratories, Inc. USA) or anti-mouse (1/100, Vector Laboratories, Inc. USA) secondary antibodies, respectively. After further washing, the sample was incubated with an avidin-peroxidase enzyme conjugate (Extravidin-peroxidase, Sigma-Aldrich, MO, USA) diluted 1:100 in PBS for 30 min at room tem-

perature. It was washed again in PBS and 0.1 M sodium acetate. The development was carried out in a mixture of a solution with diaminobenzidine 0.1% in distilled water and a solution with ammonium-nickel sulfate 5%, ClNH4 0.08% and 0.4% glucose in 0.2 M ammonium acetate. Finally, washes were performed in 0.1 M acetate and PBS, the tissue was dehydrated with 30 s passages through 70%, 96%, 100% alcohol and xylene, and mounted with Canada balsam. In the controls, incubation with primary antibodies was omitted. A morphometric study of 50 microscopy images (200X) per specimen was performed for α-SMA quantification using ImageJ software expressed as percentage of positive area. Similarly, the number of positive PCNA cells per field was calculated using 200X photography (50 images/sample) and the CellProfiler computer system (www.cellprofiler.com). The value was expressed as the mean number of cells per field.

References

1. Fiore, E.; Dominguez, L. M.; Bayo, J.; Malvicini, M.; Atorrasagasti, C.; Rodriguez, M.; Cantero, M. J.; Garcia, M.; Yannarelli, G.; Mazzolini, G., Human umbilical cord perivascular cells-derived extracellular vesicles mediate the transfer of IGF-I to the liver and ameliorate hepatic fibrogenesis in mice. *Gene Ther* **2020,** *27* (1-2), 62-73.
2. Arab, T.; Mallick, E. R.; Huang, Y.; Dong, L.; Liao, Z.; Zhao, Z.; Gololobova, O.; Smith, B.; Haughey, N. J.; Pienta, K. J.; Slusher, B. S.; Tarwater, P. M.; Tosar, J. P.; Zivkovic, A. M.; Vreeland, W. N.; Paulaitis, M. E.; Witwer, K. W., Characterization of extracellular vesicles and synthetic nanoparticles with four orthogonal single-particle analysis platforms. *Journal of extracellular vesicles* **2021,** *10* (6), e12079.
3. Pacienza, N.; Lee, R. H.; Bae, E. H.; Kim, D. K.; Liu, Q.; Prockop, D. J.; Yannarelli, G., In Vitro Macrophage Assay Predicts the In Vivo Anti-inflammatory Potential of Exosomes from Human Mesenchymal Stromal Cells. *Molecular therapy. Methods & clinical development* **2019,** *13*, 67-76.
4. Bayo, J.; Fiore, E. J.; Dominguez, L. M.; Cantero, M. J.; Ciarlantini, M. S.; Malvicini, M.; Atorrasagasti, C.; Garcia, M. G.; Rossi, M.; Cavasotto, C.; Martinez, E.; Comin, J.; Mazzolini, G. D., Bioinformatic analysis of RHO family of GTPases identifies RAC1 pharmacological inhibition as a new therapeutic strategy for hepatocellular carcinoma. *Gut* **2021,** *70* (7), 1362-1374.
